# Lifelong learning in active ageing discourse: its conserving effect on wellbeing, health and vulnerability

**DOI:** 10.1017/S0144686X16001136

**Published:** 2016-11-21

**Authors:** MIYA NARUSHIMA, JIAN LIU, NAOMI DIESTELKAMP

**Affiliations:** * Department of Health Sciences, Brock University, Ontario, Canada.; † Independent Researcher, Canada.

**Keywords:** lifelong learning, active ageing, psychological wellbeing, vulnerability, compensatory strategy, health promotion, ageing in place

## Abstract

The Active Ageing Framework has been adapted as a global strategy in ageing policies, practices and research over the last decade. Lifelong learning, however, has not been fully integrated into this discourse. Using survey data provided by 416 adults (aged 60 years and above) enrolled in non-formal general-interest courses in a public continuing education programme in Canada, this study examined the association between older adults’ duration of participation in the courses and their level of psychological wellbeing, while taking their age, gender, self-rated health and vulnerability level into consideration. An analytical framework was developed based on the literature of old-age vulnerabilities and the benefits of lifelong learning. Two logistic regression and trend analyses were conducted. The results indicate that older adults’ participation is independently and positively associated with their psychological wellbeing, even among those typically classified as ‘vulnerable’. This result provides additional evidence that suggests the *continuous participation* in non-formal lifelong learning may help sustain older adults’ psychological wellbeing. It provides older learners, even those who are most vulnerable, with a compensatory strategy to strengthen their reserve capacities, allowing them to be autonomous and fulfilled in their everyday life. The result of this study highlights the value of the strategic and unequivocal promotion of community-based non-formal lifelong learning opportunities for developing inclusive, equitable and caring active ageing societies.

## Introduction

It has been nearly 15 years since the World Health Organization (WHO) endorsed the Active Ageing Framework (hereafter the active ageing framework) in 2002. It has influenced ageing policies and practices across the world, and drawn public attention to the new opportunities and challenges that global ageing brings to both individuals and societies. The active ageing framework advocates optimising opportunities for ‘health’, ‘participation’ and ‘security’ – three key determinants of the quality of later life. It also advocates recognising physical health, mental health and social connections as equally important elements (WHO 2002). Active ageing discourse is a product of growing governmental concerns about financing health care and social services for ageing populations. At the same time, it mirrors and potentially promotes a positive shift in social attitudes towards old age: viewing later life not as a period of ‘deficits’, but as either a time of ‘competency and knowledge’ (Boudiny 2013: 1078) or a ‘period of opportunity and wellbeing, with retention or development of the psychological and cognitive resources to cope with life's challenges’ (Bowling and Iliffe 2011: 13).

Following this global trend, Canada has started working on active ageing policies, encouraging all level of governments to create age-friendly communities by improving services, programmes and infrastructure for ‘ageing in place’ (Federation of Canadian Municipalities 2013). Senior citizens (aged 65 and older) currently make up 15 per cent of the Canadian population, much smaller than other world regions like Japan and Western Europe. Yet, they are the fastest growing age group, projected to increase by 110 per cent over the next 20 years (Federation of Canadian Municipalities 2013; Statistics Canada 2012*a*). Approximately 30 per cent of current Canadian seniors are immigrants, most of who came from Europe and Asia, and have lived in Canada for several decades (Turcotte and Schellenberg 2007). It is predicted that successive cohorts of seniors will be increasingly ethno-culturally diverse (Statistics Canada 2016). Although 92 per cent of Canadian seniors live in private households, three-quarters reported at least one chronic health condition and one-quarter reported more than three conditions (Canadian Institute for Health Information 2011). Moreover, one in four seniors are living alone (Federation of Canadian Municipalities 2013; Statistics Canada 2012*b*), and 30 per cent of older Canadian women live in poverty (National Seniors Strategy n.d.). These facts suggest that one important task for seniors is to find the means to optimise wellbeing to help cope with chronic health conditions, loneliness, stress and other setbacks (Zarit 2009).

Lifelong learning in late adulthood deserves more attention in the current discourse of active ageing policies. The active ageing framework (WHO 2002) acknowledges that lifelong learning, along with formal education and literacy, is an important factor that facilitates participation, health and security as people grow older. While a few places, such as the European Commission (Oxley 2009) and the Province of British Columbia, Canada (Government of British Columbia n.d.), have explicitly inscribed ‘lifelong’ in their active ageing policies, generally speaking, it is mentioned far less often than topics like physical activities and paid and non-paid work. Nevertheless, an increasing body of literature has found clear links between adult learning and learners’ subjective wellbeing and health (Dolan, Fujiwara and Metcalfe 2012; Feinstein and Hammond 2004; Feinstein *et al.* 2008; Field 2009, 2011; Hammond and Feinstein 2006; Manninen *et al*. 2014; Schuller *et al*. 2004), while irrefutable evidence exists to show that these benefits are even greater for vulnerable groups (Bailey, Breen and Ward 2010; Dolan, Fujiwara and Metcalfe 2012; Feinstein *et al.* 2008; Manninen *et al*. 2014).

Studies that directly examine the effects of later-life learning on older adults, however, are few and far between. In many countries, adults over the age of 65 are often excluded from adult learning statistics as they are assumed to be the post-work generation. In addition, research in adult learning traditionally focuses more on ‘transforming effects’ (*e.g.* personal change, community activism) than ‘conserving effects’ (*e.g.* self-maintenance, the social fabric), where ‘education prevents decay or collapse or consolidates a positive state of stability’ (Schuller 2004: 25). Given the context of global ageing, it becomes more important to investigate the latter effects of learning, which can be quite invisible, yet play an important role in maintaining autonomy, health and quality of life among older adults. Although still limited in volume, recent studies examining the association between later-life learning and psychological wellbeing suggest that continued engagement may help older learners sustain wellbeing over the long term (Jenkins 2011; Jenkins and Mostafa 2015; Leung and Liu 2011; Narushima 2008). The purpose of this study, therefore, is to identify further the effect of the duration of older adults’ participation in lifelong learning on their psychological wellbeing, while taking into consideration their health state, vulnerability and other key demographic variables.

## Literature review

### Active ageing, health and chronic conditions

The idea of being active for a good old age is not new. However, the principle of the WHO's (2002) active ageing framework differs from traditional activity theory (Havighurst 1961; Neugarten, Havighurst and Tobin 1968). Activity theory has been criticised for its unrealistic expectation that older adults can maintain the same levels of activity as they did in middle age by ‘denying the onset of old age’ (Walker 2002: 122). The active ageing framework advocates a broader approach, which defines ‘being active’ as continuing to engage in *any* social, economic, cultural or civic activity at the level of one's capacity (Boudiny 2013). The idea is to embrace not only the individual's right and responsibility to remain active, but also the government's responsibility to create an age-friendly social system and community environment. Consequently, in theory, the framework calls for all levels of governments to take action to ensure opportunities for all older adults, especially those who are most vulnerable, to stay involved, healthy and secure (Boudiny 2013; Mayhew 2005; Walker 2002, 2006; WHO 2002).

Nonetheless, the adaptation of this framework is far from flawless. Boudiny (2013) in the United Kingdom (UK) argues that the active ageing framework tends to be narrowly used to promote physical activity and prolonged labour participation, so that it is often used interchangeably with ‘productive ageing’ and ‘healthy ageing’. According to Boudiny, this dominant discourse risks falling into the same pattern as activity theory did a half century ago, because it posits health and independence – unattainable for vulnerable adults who are frail, dependent and very old – as its ultimate goal. In order to develop more inclusive policies and practices, she proposes an alternative vision that acknowledges health as a resource for and an outcome of active ageing, not as a prerequisite for or a goal of active ageing (Boudiny 2013).

Recent research has started paying attention to the experiences of active ageing with chronic conditions. Richardson, Grime and Ong's (2014) qualitative study in the UK found that their participants (N = 27, aged 55–90), who lived with severe osteoarthritis, pragmatically just ‘kept going’ in body and mind despite chronic pain and stiffness. Lassen's (2015) fieldwork in two activity centres in Denmark also found that his participants (N = 17, aged 58–92) enjoyed various activities despite a range of serious chronic conditions, thereby ‘keeping disease at arm's length’. Lassen (2015: 1367) argues that engagement in ‘social and/or physical activity forms enough distance to help older adults focus on wellness rather than illness, and manage their everyday life to delay further physical deterioration’. What these studies (Boudiny 2013; Lassen 2015; Richardson, Grime and Ong 2014) share is a realistic picture of growing old, one that acknowledges the changes and challenges of late adulthood.

### Vulnerabilities, reserve capacities and compensatory strategies in later life

The notion of ‘vulnerabilities’ provides a helpful basis to analyse the roles of lifelong learning in the light of active ageing. Vulnerabilities in old age involve various ‘risks’ that may cause a poor quality of later life or premature or unintended death (Grundy 2006; Schroder-Butterfill and Marianti 2006). According to the ‘old-age vulnerability framework’, the most high-risk sub-groups of older adults are the old-old, those with low incomes, those with limited education, those widowed and living alone, those with poor social networks, those in poor health and women (Grundy 2006; Schroder-Butterfill and Marianti 2006). In other words, vulnerabilities in late adulthood are determined by individuals’ socio-demographic characteristics (*i.e.* age, income, education level, marital status, gender), their living and health conditions, the availability of resources and their ability to use those resources. It is reasonable to assume that the more risk factors, the more likely an older person will suffer severe outcomes, as the risks are more likely to intersect.

Nonetheless, according to the vulnerabilities framework, negative consequences arise only when the balance between a person's risk factors/conditions and his or her reserve capacities falls below his or her control to ensure a reasonable quality of life (Grundy 2006; Schroder-Butterfill and Marianti 2006). Vulnerability involves four domains: ‘states’ (*i.e.* personal characteristics), ‘threat’ (*i.e.* events/environmental challenges), ‘coping capacity’ (*i.e.* an individual's reserve capacities such as positive wellbeing, social network and utilisation of compensatory strategies) and ‘outcome’ (*i.e.* quality of life and survival). The interaction of the first three domains determines the fourth: *i.e.* either a negative or positive outcome (Grundy 2006). Consequently, not every older adult who shares similar risk factors/conditions ends up with a negative outcome. It depends on one's reserve capacities and how one can utilise compensatory strategies to change and manage life (Schroder-Butterfill and Marianti 2006). While reserve capacities and compensatory strategies in later life are largely influenced by a person's life history and social environment, this does not mean that it is impossible to reinforce or develop them in old age. According to the old-age vulnerabilities framework, it is important to recognise the significant effects of *current* circumstances and behaviour on the general wellbeing of older people (Grundy 2006: 108). In this study, we thus view the participation in a specific form of lifelong learning programme as a compensatory strategy that older adults can draw on to improve those reserve capacities.

### Psychological wellbeing as a predictor of health and quality of life

Psychological wellbeing is one of the most important reserve capacities. While its definition differs slightly from study to study (Diener 2006; Olsson *et al*. 2013), psychological wellbeing generally involves the individual's subjective evaluation of various aspects of his or her life (*e.g.* of events, physical and mental issues, and life circumstances). It consists of three basic elements: the individual's *affect* (*e.g.* positive or negative emotional states), *ability* (*e.g.* adaptive capacity or coping skills) and *perception* (*e.g.* satisfaction, purpose and outlook) (Catolico 1997, cited in Momtaz *et al*. 2011; Diener 2006; Organisation for Economic Co-operation and Development (OECD) 2013; Olsson *et al*. 2013; Ryff *et al*. 2006). Psychological wellbeing as used in this study is closely related to concepts such as eudaimonic wellbeing, positive functioning and self-actualisation, which entails aspects of life satisfaction, autonomy, integrity, personal growth, purpose of life, competence and mastery, and positive relatedness (Ryan and Deci 2000; Ryff and Keyes 1995, cited in Vanhoutte 2014).

Psychological wellbeing in late adulthood is a complex variable which may be influenced by both personal as well as social factors including: the older individual's demographic characteristics (*e.g.* age, marital status, education level, occupation and income, degree of social integration and social support), health state (*e.g.* self-rated health, chronic diseases, functional capacity), availability of supportive social environment and cultural values in their society (*e.g.* ageism) (Iwasa *et al*. 2006; Momtaz *et al*. 2011; Olsson *et al*. 2013; Vanhoutte 2014). Not surprisingly, the personal and social factors affecting psychological wellbeing overlap with the aforementioned risk factors and vulnerabilities in later life. It is thus reasonable to assume that the higher the degree of vulnerability, the more chances an older person will report a lower level of psychological wellbeing.

Since these risk factors and conditions are related to mortality, psychological wellbeing is also used as a relevant predictor for physical health, survival and quality of life in later adulthood (Iwasa *et al*. 2006). A number of studies in psychology and health have found that a higher level of psychological wellbeing leads to better coping with life events (Andrews 2001), lower morbidity, decreased symptoms and pain (Pressman and Cohen 2005), a lowered risk of cardiovascular disease and distress (Ryff *et al*. 2006) and reduced falling (Anstey *et al*. 2008). Conversely, a higher level of ill-being (*e.g.* anxiety, anger, depressive symptoms) is a predictor of an increased risk of functional disability (Cuijpers, de Graaf and van Dorsselaer 2004), cardiovascular disease (Ryff *et al*. 2006) and mortality (Howell 2009; Iwasa *et al*. 2006).

Combined, these studies confirm the importance of maintaining positive psychological wellbeing as a reserve capacity for health and quality of life to help mitigate unwanted outcomes in the face of older adults’ vulnerabilities. They also suggest why we should examine psychological wellbeing not only in terms of individuals’ efforts for emotional regulation, but also as it is related to social contexts, including the availability of compensatory strategies to maintain positive wellbeing.

### Benefits of lifelong learning participation

Over the last decade, a growing body of literature in adult education has found psychological wellbeing and health to be an important part of the wider benefits of lifelong learning. Large-scale studies in the UK and Europe have unanimously supported the positive link between lifelong learning participation and psychological wellbeing and health among adult learners (Dolan, Fujiwara and Metcalfe 2012; Feinstein and Hammond 2004; Feinstein *et al.* 2008; Field 2009, 2011; Manninen *et al*. 2014; Schuller *et al*. 2004).

Although the complex pathways linking learning participation and positive wellbeing and health require more study, Hammond (2004) in the UK has provided a helpful conceptualisation of psychological resources as the mediators between adult learning and health outcomes. Based on her interviews (N = 145, aged 16–70), she found that learning helps adults develop the five psycho-social resources (*i.e.* ‘self-esteem and self-efficacy’, ‘identity’, ‘purpose and hope’, ‘competences and communication’ and ‘social integration’) that promote their general ‘wellbeing’, ‘mental health’ and ‘effective cop(ing) with change and adversity including ill health’ (Hammond 2004: 40). These findings clearly suggest that participating in adult learning courses functions as a compensatory strategy for adults to help them develop psychological and social reserve capacities.

Other researchers who have focused on later-life learning see this point as especially relevant to ‘non-formal’ and ‘non-credit’ types of learning. Narushima (2008) conducted interviews (N = 15, aged 64–83) and observations of five different general-interest courses for older adults (calligraphy, sewing, Chinese poetry, folk dance and fitness) in a public continuing education programme in Canada. She found that weekly engagement in the course fuels older learners with intrinsic motivations to keep going physically and mentally, providing joy and satisfaction, a sense of self-growth and mastery, and friendships with classmates and teachers. As with Richardson, Grime and Ong (2014) and Lassen (2015), all interviewees in Narushima's (2008) study had some chronic conditions, and consciously pursued their weekly classes and related learning practice at home as part of their health maintenance and self-management practice. In the UK, based on focus groups (N = 98), questionnaires (N = 77) and follow-up interviews (N = 35), Withnall (2010) found many of her post-work participants regarded their learning in retirement as keeping their minds active, gaining new knowledge, relaxation and increased social contact.

More recent quantitative studies have further examined the association between participation in later-life learning and psychological wellbeing. Jenkins and his colleague examined the long-term effects of different types of lifelong learning on the psychological wellbeing of British older adults, following more than 3,000 older adults between 2002 and 2009 with data from the English Longitudinal Study of Ageing (Jenkins 2011; Jenkins and Mostafa 2013, 2015). They found that while higher levels of education are generally related to higher levels of wellbeing, without intervention there is a gradual decline in older adults’ psychological wellbeing over time, regardless of their level of education (Jenkins and Mostafa 2013). However, they also found that, after controlling for socio-economic characteristics and other unobserved factors, participation in non-formal and non-credit type learning (*e.g.* music/arts/evening classes, sports club/exercise classes) was especially beneficial to maintain older adults’ psychological wellbeing. The participation in these non-formal learning courses raised older adults’ psychological wellbeing by between 4 and 11 per cent of a standard deviation, while formal education and training courses were not associated with higher wellbeing. The authors concluded that more informal, leisure-type courses were most likely to sustain older adults’ participation, consequently enhancing their wellbeing over time (Jenkins 2011; Jenkins and Mostafa 2013).

In a similar vein, Leung and Liu (2011) in China undertook a cross-sectional study to identify the relationship between participation in lifelong learning, quality of life and the self-efficacy of older learners (N = 1,003). While they found overall benefits involving older adults’ participation in learning and their quality of life, they also found that older learners who reported a higher level of quality of life were not those who enrolled in many courses at one time, but rather those who participated in a single course continuously. The authors conclude that what significantly influences the quality of life among older adults is not the amount, but the continuation of learning and the maintenance of self-efficacy.

Following these studies, Narushima, Liu and Diestelkamp (2013*a*) went back to the previously studied public continuing education programme (non-credit general-interest courses) in Canada (Narushima 2008) to undertake a cross-sectional survey with 699 older learners (aged 60 and older) enrolled in order to investigate whether what had been found in the previous qualitative study (Naruushima 2008) could be generalised programme wide. The results showed that older learners generally reported positive wellbeing and healthy lifestyles such as non-smoking and regular exercise. The result also indicated a positive association between the duration of participation and overall levels of wellbeing after controlling for key covariates such as socio-demographic characteristics and health-related factors (Narushima, Liu and Diestelkamp 2013*b*).

These existing studies suggest the positive effects of continuous participation in lifelong learning programmes on psychological wellbeing. Nonetheless, they still leave some questions unanswered. In particular, when examining the effects of duration, selective attrition (*i.e.* those who feel better and healthy tend to remain in the programme) is one of the questions. Can older adults facing multiple risk factors/conditions keep participating in and benefiting from these lifelong learning programmes? The present study approaches this question by examining whether the positive association between duration of participation and older learners’ psychological wellbeing remains even after levels of vulnerability and multiple risks have been taken into consideration.

## Analytical framework

**Figure 1.**
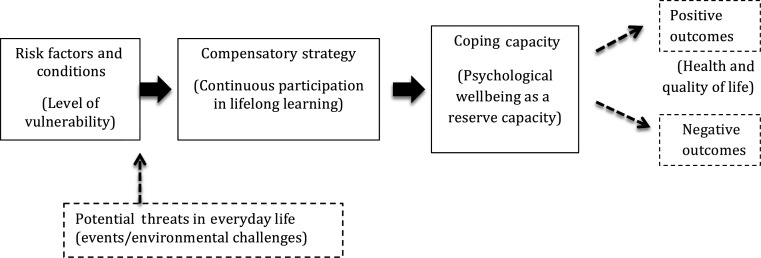
The effects of lifelong learning on wellbeing, health and vulnerabilities in later life.

Bringing together the old-age vulnerabilities framework and the benefits of lifelong learning, we developed a conceptual framework for analysis in this study (Figure 1).

Our framework includes potential ‘threats’ and ‘outcomes’ (presented in dotted squares and arrows), following the old-age vulnerabilities framework (Schroder-Butterfill and Marianti 2006). In this study, however, we only focused on the associations between ‘risk factors/conditions’, ‘compensatory strategy’ and ‘coping capacity’, which were operationalised as the ‘level of vulnerability’, ‘continuous participation in a lifelong learning programme’ (*i.e.* duration) and ‘psychological wellbeing as a reserve capacity’.

## Methodology

### Sampling group

Older learners aged 60 and above, enrolled in a public non-formal continuing education programme during the fall of 2010, were targeted with a programme-wide survey. The programme was run by a local school board, which partnered with various community facilities such as community centres, adult learning centres, high schools and retirement homes. It offered non-credit general-interest courses in four subject areas: (a) arts and crafts, (b) fitness and exercise, (c) music and dance, and (d) language, computer and other practical skills. Four research assistants visited 158 classes and distributed a self-administered survey that asked for three types of information (*i.e.* basic demographic and health-related information, general psychological wellbeing and participation patterns). The questionnaire was completed at home by participants and returned the following week in class. Ethics clearance was received from the research ethics boards of the researchers’ university and the school board.

In total, 1,331 older learners were approached with 921 (69.20%) responding. Of the 921, 416 individuals provided consent forms with no missing data on the variables necessary for the models of analysis that were included for this study. Since participants were recruited from one of the largest public continuing education programmes in a large metropolis, we had a diverse sample group. We thus further divided the participants into three groups (*i.e.* high risk, middle risk and low risk) based on their socio-demographic, behavioural and health states.

### Measurements

#### Psychological wellbeing

The older adults’ psychological wellbeing during the previous six months was measured by the 22-item Psychological General Well-Being Index (PGWBI) (Dupuy 1984, 2004). The PGWBI is reliable and has been validated by numerous studies (Dupuy 2004; Grossi *et al*. 2006; McDowell 2006; Nilsson *et al*. 2011; Wu 2004). It uses six dimensions to measure psychological wellbeing, including both positive and negative attributes: (a) depressive moods, (b) anxiety and stress, (c) self-control, (d) concerns about general health, (e) life satisfaction and (f) vitality (Dupuy 2004; McDowell 2006). Answers are assigned scores from 0 to 5, and then used to make dimensional scores as well as a global score. The global wellbeing score is divided into three levels: ‘severely distressed’ (0–60), ‘moderately distressed’ (61–72) and ‘positive wellbeing’ (73–110) – the higher the score the higher the level of wellbeing. In order to examine whether continued participation in a local public lifelong learning programme might help maintain older adults’ psychological wellbeing, we created a dichotomous variable by classifying the participants’ wellbeing status into two categories (*i.e.* ‘distressed’ when the global wellbeing score was <73, and ‘positive wellbeing’ when the score was 73 or higher). This variable was the dependent variable in logistic regression analysis.

#### Vulnerability level

In order to reflect a participant's vulnerability level, we created an index vulnerability level score by using information obtained from the survey. This index was comprised of age and indicators of three domains (*i.e.* health, socio-economic status and social support), with a higher index score reflecting a higher level of vulnerability or risk. Health indicators included whether the person had chronic conditions (*e.g.* diabetes, high blood pressure, arthritis), difficulties in daily living (*e.g.* walking, climbing stairs, hearing), and whether he or she regularly exercised for at least 20 minutes a day. Socio-economic status indicators included income and education level, while social support indicators included marital status, number of confidants, living arrangements and whether an individual participated in other social activities. An overall breakdown of how the vulnerability index variable was created is shown in Table 1.

**Table 1.** Vulnerability index variable composition

Indicator type	Sub-variable name	Risk condition value
Health indicator	Chronic diseases	>= 3 conditions = 3
		2 conditions = 2
		1 condition = 1
		0 conditions = 0
	Difficulties in daily life	>= 3 difficulties = 3
		2 difficulties = 2
		1 difficulty = 1
		0 difficulties = 0
	Participants in regular exercise	Yes = 0
		No = 1
Socio-economic status indicator	Yearly income	<=Can $25,000 = 3
		Can $25,001–45,000 = 2
		Can $45,001–65,000 = 1
		>=Can $65,001 = 0
	Highest educational level	Primary school = 2
		Secondary school = 1
		University/college = 0
		Other = 0
Age indicator	Age	85 and older = 2
		75–84 years = 1
		64–74 years = 0
Social support indicator	Marital status	Married = 0
		Not currently married = 1
	Number of confidants	5 or more people = 0
		2–4 people = 1
		1 or 0 people = 2
	Living arrangements	Alone = 1
		With someone else = 0
	Participates in other social	Yes = 0
	activities^1^	No = 1

*Note*: 1. Includes any other group-organised activities such as clubs, associations, religious organisations, volunteering, recreational activities, *etc*.

Components of the index of the vulnerability level variable were chosen based on results from univariate analyses and the old-age vulnerabilities framework (Grundy 2006; Schroder-Butterfill and Marianti 2006). Each explanatory variable comprising the index was given a score; these scores were then added together to give an overall score for each participant. The average vulnerability level score of participants was 5.18, while the median score was 5.0 (a higher score indicates higher vulnerability). Participants were then divided into three categorical groups based on their overall score: low risk (0–4; n = 175), middle risk (5 or 6; n = 129) and high risk (7 or higher; n = 112).

#### Self-rated health

In addition to the health indicators in the index of the vulnerability level, we also asked older adults to rate their general state of health by asking the question: ‘Compared with other people your age, how would you say your general health is?’ The choice of answers included: excellent, very good, good, fair and poor. The fair and poor groups were joined during analysis to ensure the size of each of the groups remained roughly similar.

#### Duration of learning

Older learners’ participation patterns, including learning duration, were gathered through the final section of the survey. The duration of class participation was examined by asking participants how many years and months they had taken the course. For the purpose of this study, those who had participated for three months or less (*i.e.* those in their first term) were excluded as they were still likely adjusting to the programme. The time of duration was then split into three groups: 4–18 months (short duration group), 19–48 months (middle duration group) and 49 months and longer (long duration group).

### Statistical analysis

Chi-square, analysis of variance and *t*-tests were performed to examine whether there were differences in frequency distributions or averages. Potential confounders included gender, age, vulnerability level and self-perceived health. Two different logistic regression models were used to examine the association between duration and wellbeing. For both models, the dependent variable was the overall wellbeing status (1: the global PGWBI score ⩾73; 0: <73, higher values of PGWBI indicate better wellbeing) and the predictive variable was learning duration. The first model examined the association between duration and wellbeing, while taking into account the covariates of age, gender and vulnerability level. The second model included age and gender, but the vulnerability level was replaced with self-perceived health, since they were strongly correlated. Following logistic regression, trend analysis was performed for each model. All data were analysed using SAS 9.2.2 with the significant level set at equal or less than 0.05 on two-tailed tests.

## Results

Table 2 shows the characteristics of each of the three duration groups. Of the 416 participants included in this study, the majority of participants in each duration group were female (74.5% in the short duration, 77.3% in the middle duration and 74.0% in the long duration group). Participants in each group had similar characteristics except that individuals in the long duration group were older and had a higher proportion positive wellbeing.

**Table 2.** Characteristics of participants by duration of class participation

	Short duration (4–18 months)	Middle duration (19–48 months)	Long duration (49+ months)	*p*
n (%)	106 (25.5)	141 (33.9)	169 (40.6)	–
Mean age (SD)^1^	67.2 (5.4)	68.4 (4.9)	73.0 (7.1)	<0.001
Females (%)	74.5	77.3	74.0	0.779
	*Percentages*			
Education status:^1^				0.228
Primary	1.0	1.4	3.6	
Secondary	17.9	17.1	19.1	
College/university	74.5	71.6	62.9	
Other	6.6	9.9	14.4	
Marital status:^1^				0.136
Married	53.8	56.4	55.6	
Separated	2.9	2.2	3.6	
Divorced	19.8	13.6	7.1	
Widowed	9.4	16.4	18.3	
Single	11.3	10.7	13.6	
Other	2.8	0.7	1.8	
Total income level (Can $):^1^				0.350
<=25,000	24.2	19.1	19.2	
25,001–45,000	30.3	20.6	24.4	
45,001–65,000	18.2	15.3	19.2	
>=65,001	14.2	22.1	16.7	
Prefer not to reveal	13.1	22.9	20.5	
Self-perceived health:				0.250
Excellent	25.5	17.7	18.9	
Very good	37.7	46.8	44.4	
Good	27.3	29.1	33.1	
Fair/poor	9.4	6.4	3.6	
Living arrangement:^1^				0.059
Alone	30.2	31.9	37.5	
Spouse	50.0	53.9	56.0	
Spouse and other	3.8	3.6	0.6	
Other	16.0	10.6	5.9	
Mean number of confidants (SD)^1^	5.5 (6.2)	5.8 (6.9)	5.3 (4.2)	0.563
Participates in exercise:^1^				0.137
No	17.8	16.4	25.0	
Yes	82.2	83.6	75.0	
Vulnerability levels:				0.066
Low (0–4 risks)	36.8	47.5	40.8	
Middle (5 or 6 risks)	34.9	33.3	26.6	
High (7+ risks)	28.3	19.2	32.6	
Psychological wellbeing:				0.027
Distressed	21.7	12.1	10.7	
Positive wellbeing	78.3	87.9	89.3	

*Notes*: SD: standard deviation. Sample size may vary for some variables due to missing data. 1. Missing values: Age: 8; Education: 2; Marital status: 1; Income level: 30; Living arrangement: 1; Confidants: 22; Regular exercise: 7.

The overall results of both logistic regression models can be seen in Tables 3 and 4. In Model 2, as seen in Table 3, those who had a higher level of vulnerability were more likely to be distressed than those with lower levels of vulnerability. Compared to those with vulnerability index scores of 0–4, the odds ratio (OR; 95% confidence interval (CI)) of being distressed was 3.2 (1.3–7.8, *p* = 0.0102) for those with index scores of 5–6 and 8.8 (3.7–20.9, *p* < 0.0001) for those with index scores of 7 or more. Similarly, Table 4 shows that an individual reported lower self-perceived health, the OR (95% CI) of being distressed was 3.8 (0.8–17.1, *p* = 0.0867), 11.9 (2.7–52.4, *p* = 0.0011), and 57.2 (11.0–296.7, *p* < .0001) for those in very good, good and fair/poor groups, respectively when compared to those with excellent health.

**Table 3.** Odds ratios of distressed (Model 1)

Model	Odds ratio	95% confidence interval
Gender	0.936	0.467–1.877
Age	0.993	0.943–1.046
Duration:		
4–18 months	1.000	–
19–48 months	0.617	0.298–1.278
49+ months	0.399	0.181–0.881
Risk group:		
0–4 risks	1.000	–
5 or 6 risks	3.204	1.317–7.794
⩾7 risks	8.788	3.699–20.878

**Table 4.** Odds ratios of distressed (Model 2)

Model	Odds ratio	95% confidence interval
Gender	0.967	0.471–1.984
Age	1.006	0.955–1.060
Duration:		
4–18 months	1.000	–
19–48 months	0.474	0.221–1.019
49+ months	0.405	0.180–0.914
Self-perceived health level:		
Excellent	1.000	–
Very good	3.756	0.827–17.064
Good	11.892	2.699–52.391
Fair/poor	57.160	11.012–296.707

**Figure 2.**
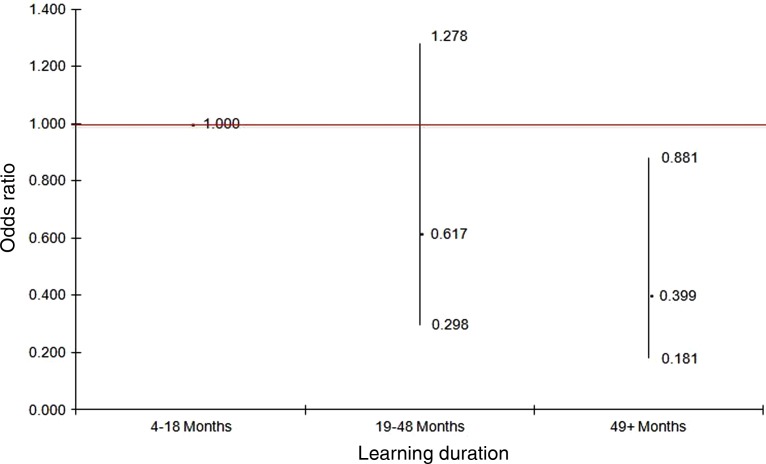
Odds ratios and 95 per cent confidence intervals of distressed for different learning durations after adjusting for gender (female *versus* male), age (years) and risk group (5 or 6 risks and 7 or more risks *versus* 0–4 risks) (Model 1).

**Figure 3.**
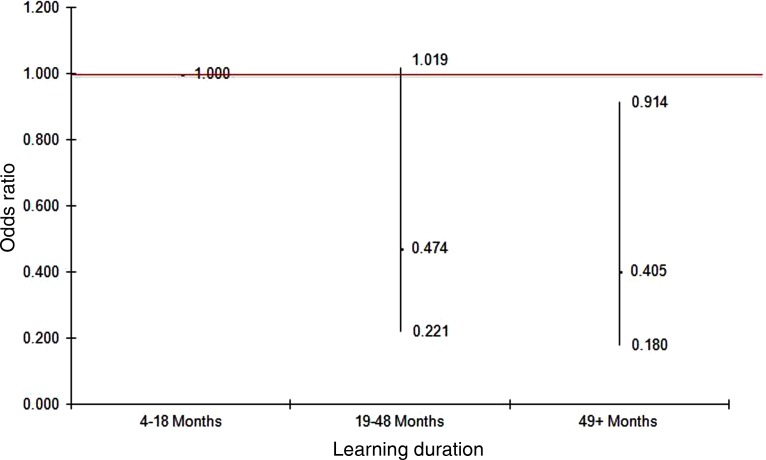
Odds ratios and 95 per cent confidence intervals of distressed for different learning durations after adjusting for gender (female *versus* male), age (years) and self-perceived health (very good, good and fair/poor *versus* excellent) (Model 2).

Figures 2 and 3 provide OR (and 95% CI) for the duration variable from the logistic regression analyses. Figure 2 shows the adjusted OR of being distressed by learning duration when taking into account age, gender and vulnerability index score; while Figure 3 shows the adjusted OR by learning duration when taking into account age, gender and self-perceived health.

*Significance level:p* < 0.03 (for trends).

*Significance level:p* < 0.03 (for trends).

When examining the association between an older individual's learning duration and their general psychological wellbeing in comparison with those in the shortest duration group, the odds of being in the distressed category for those in the longest duration group decreased 60 per cent after adjusting for age, gender and overall vulnerability index score (OR = 0.4, 95% CI = 0.2–0.9, *p* = 0.02). Similar results were observed in Model 2 when self-perceived health was taken into account (*p*-values for trends in both models were less than 0.05).

## Discussion

The results of this study indicated a clear association between continuous participation in the specific form of lifelong learning courses and the psychological wellbeing of older adults, even after controlling for age, gender, health state and vulnerabilities. The findings generally support the benefits of meaningful social participation for active ageing and enhanced quality of life in old age (Adams, Leibbrandt and Moon 2011; Butler 2002; Gilmour 2012). More specifically, this study provides additional evidence for the benefits of lifelong learning on older adults’ psychological wellbeing and health (Dench and Regan 2000; Jenkins 2011; Jenkins and Mostafa 2013, 2015; Leung and Liu 2011; Narushima 2008; Narushima, Liu and Diestelkamp 2013*a*; Preece and Findsen 2007; Withnall 2010).

The results showed that the longer older adults remained on one course or pursued the same subject, the better psychological wellbeing they reported, even after all key covariates were taken into account. *Continuous and ongoing* participation in lifelong learning programmes contribute to the conservation of older learners’ psychological wellbeing over time. This point is noteworthy from the perspective of old-age vulnerability, as it confirms what Grundy (2006: 108) articulated as the ‘very important effect of *current* circumstances and behaviour’ on the wellbeing of older adults.

In fact, an unexpected finding of our study is that educational level did not play a significant role in influencing the level of wellbeing in either of the two logistic regression models, despite the accumulated evidence of the lifelong effects of formal education for an individual's wellbeing and health (Mirowsky and Ross 2005, 2008). One possible reason might be that both the direct and indirect effects of schooling at a young age on health and wellbeing change across the lifecourse. A study found that the schooling–income effect increases until age 60, plateaus and then declines after age 80 (Lynch 2006). Another study found that average health–literacy scores consistently fall as people age, regardless of their socio-economic status (Will, Douglas and Murray 2007, cited in Murray *et al*. 2008). The aforementioned longitudinal study of British older adults (Jenkins and Mostafa 2013) also found that one's psychological wellbeing gradually and equally declines over the years regardless of one's educational background. Bringing together all these studies, one can say that, in order to sustain psychological wellbeing – an important reserve capacity – and avoid the negative consequences of vulnerabilities in old age, older people need to take some action to compensate for their decline, regardless of their degree of vulnerability. The result of the present study corroborates that the continuous participation in a lifelong learning course is one effective compensatory strategy.

In addition, the results of this study highlighted the protective effect of a specific form of lifelong learning for older adults, particularly those most vulnerable. In contrast to our hypothesis that the longer the participation, the lower the vulnerability levels due to selective attrition, nearly one-third of our participants in the longest duration group fell into the ‘high-risk’ category (*i.e.* with more than seven risk factors/conditions). Yet, members of that group reported equally increased psychological wellbeing regardless of that vulnerability. This is probably because their local, non-formal and non-credit leisure and recreational type of lifelong learning programme, with its supportive environment, can enable even the most vulnerable and frail older adults to continue to participate in their active ageing lifestyles at their own pace (Lassen 2015; Richardson, Grime and Ong 2014). This in turn helps restore their psychological reserves including wellbeing, motivating their participation and maintaining their coping capacity, while mitigating the effects of their risk conditions (Grundy 2006; Hammond 2004; Schroder-Butterfill and Marianti 2006).

Aligned with other studies that take a more inclusive and broader view of active ageing (Boudiny 2013; Lassen 2015; Richardson, Grime and Ong 2014), participants in this study also demonstrated that physical health is not a prerequisite for an active lifestyle in old age. Nonetheless, given the empirical link between a higher level of wellbeing and better physical function and health (Andrews 2001; Anstey *et al*. 2008; Pressman and Cohen 2005; Ryff *et al*. 2006), and, conversely, higher levels of ill-being as a predictor of increased functional disability and mortality (Cuijpers, de Graaf and van Dorsselaer 2004; Howell 2009; Iwasa *et al*. 2006; Ryff *et al*. 2006), it is quite possible that continuous engagement in later-life learning activities can create a virtuous cycle to prevent or slow down the onset of diseases and physical deterioration. This may also be why more vulnerable older adults with poorer health tend to report higher perceived benefits of and appreciation for lifelong learning programmes on their wellbeing and health (Dench and Regan 2000; Narushima, Liu and Diestelkamp 2013*b*; Manninen *et al*. 2014).

Nevertheless, the results of our study do not necessarily mean that all types of lifelong learning have a similar effect on older adults’ psychological wellbeing. Consistent with what previous studies have found (Jenkins 2011; Jenkins and Mostafa 2013, 2015; Manninen *et al*. 2014), our study showed the positive effects of non-formal and non-credit lifelong learning courses – often undervalued as leisure and recreation – on the maintenance of older adults psychological wellbeing. To be clear, the lifelong learning programme examined in this study is a publicly funded, non-formal and non-credit programme offering diverse general-interest subjects. It is also worth mentioning that all classes are easily accessible by public transportation, and that the school board subsidises fees for low-income seniors. This equity-oriented programme environment makes it easier for those with some physical and mental health conditions or lower incomes to continue to pursue their personal interests in their local communities.

Clearly, more research is needed to investigate how and why continuous participation in lifelong learning matters so much in later life. Nonetheless, combining the results of our study with the existing literature, we speculate that participation in community-based non-formal lifelong learning plays different roles at two levels. At the individual level, psychological qualities such as ‘self-esteem’, ‘self-efficacy’ and ‘resilience’ (Hammond 2004) – immediate and direct benefits of learning – may need to be more frequently reinforced in late adulthood to sustain an individual's psychological wellbeing, due to the increased need to cope with physical, material and social changes. Later-life development is a continuous process of behavioural and psychological adaptation, the regulation of one's resources through the process of ‘selection, optimisation and compensation’ (SOC) (Baltes and Baltes 1990; Freund and Baltes 1998). According to this theory, those who successfully select valued activities continue to find compensatory strategies, optimise desired outcomes, and have higher levels of emotional wellbeing and life satisfaction. Older learners who stick with their subject of interest over the years may be going through a similar process as the SOC, thereby obtaining a sense of continuity and satisfaction that compensates for the changes and consequent worries and anxieties they face in their daily lives.

At the social level, continuous participation on the same course also helps older adults develop connections and a stronger sense of community with fellow students and instructors, providing them with informal social support (Dench and Regan 2000; Narushima 2008; Schuller *et al.* 2004; Withnall 2010). The results of this study showed that there was no difference in the number of confidants despite the higher average age of the longer duration group, and the fact that the number of one's confidants and friends decreases when one gets older. As this result suggested, staying in a single programme helps older adults build and maintain an informal social support network. While not the main focus of our investigation, this result nevertheless suggests that long-term participation in community-based non-formal lifelong learning is not only of benefit for psychological wellbeing at the individual level, it also provides a ‘social fabric’ or ‘a collective environment that is conducive to sustaining health’ as advocated by Schuller *et al.* (2004: 27).

Given our goal to realise ageing in place through creating age-friendly communities within an active ageing framework (WHO 2002), this indirect benefit at the social level should be considered as an equally important aspect of the ‘conserving effects’ of community-based non-formal lifelong learning (Schuller 2004).

## Conclusion

Given the limitations of cross-sectional study design and the general methodological challenges involved in assessing the impact of lifelong learning on psychological wellbeing with its many possible confounding variables, we were unable to determine causation. Furthermore, the problem of selective attrition was not completely solved despite our creation of the three vulnerability levels, because those who participated in this survey may have had higher levels of wellbeing than those who did not. Moreover, those enrolled in this continuing education programme, to take full advantage of its benefits, may have had higher levels of wellbeing at the outset. Organised learning and educational activities may cause stress and anxiety for some older adults due to their negative experiences in formal education (Field 2009), so that such people may not participate in the programme to begin with. In addition, this study encompassed people on non-credit courses only, so no comparison can be made with those older people who take credit courses but are not engaged in lifelong learning activities.

Despite these limitations, the findings of this study signify that *continuous and ongoing participation* in a community-based non-formal lifelong learning programme may work as a compensatory strategy for older adults by helping them sustain their psychological wellbeing – an important source of support and protection for autonomy and self-management in later life. Continuing engagement in activities and relationships that they value can help older people focus on wellness rather than illness, despite chronic conditions and other challenges in later life. The role of later-life learning should be understood as far more than merely instrumental. Nevertheless, its conserving effect on wellbeing and health underscores the need for more strategic and unequivocal policies and practices to promote lifelong learning among older adults as a vital part of the active ageing framework.

Unfortunately, personal and structural barriers prevent many older adults from taking advantage of these benefits. Participation in adult learning and education is unevenly distributed between countries and within a given country. For example, in OECD countries, the participation of more educated adults is three to five times higher than those with less education – the more vulnerable group (Desjardins 2015). In addition, participation in lifelong learning (both formal and non-formal) drastically decreases with age. A survey of 14 European countries found that the average participation rate in education or training courses among those 60–69 years old was 7 per cent, of those 70 years and older only 3 percent (Kolland and Wanka 2014). Nevertheless, the same research also suggests that the participation rate varies hugely even between countries, and that Nordic countries with equity-oriented public policies have much higher participation rates (Kolland and Wanka 2014).

Given the lifelong learning divide, the decrease in participation in learning activities in later life and the ongoing budget cuts for adult learning in many countries, it is crucial to promote the benefits of lifelong learning to allow all older adults – especially those whose poor educational, financial, social or health background makes them more vulnerable – to take advantage of this effective compensatory strategy. In order to develop more inclusive and equitable active ageing policies and practices, Boudiny (2013: 1088–93) advocates three additional principles: ‘fostering adaptability’, ‘supporting the maintenance of emotionally close relationships’ and ‘removing structural barriers related to age or dependency’. Publicly subsidised non-credit general-interest lifelong learning programmes, like the programme examined in this study, can play a vital role to help realise these principles. Policy makers at all level of governments should take a fresh look at lifelong learning programmes as a necessary and cost-effective strategy for promoting wellbeing and health, and develop more affordable, accessible and diverse later-life learning programmes to achieve the goal of active ageing in place for all. Creating an active and caring ageing society, after all, is the responsibility of both individuals and governments.
